# Supplementary material for: Simultaneous Measurement and Distribution Analysis of Urinary Nicotine, Cotinine, Trans-3′-Hydroxycotinine, Nornicotine, Anabasine, and Total Nicotine Equivalents in a Large Korean Population
Source: Molecules. 2023 Nov 21;28(23):7685. doi: 10.3390/molecules28237685 (PMC10708046; doi:10.3390/molecules28237685)
Supplement: Supplementary file 1 [file molecules-28-07685-s001.zip › molecules-2658824-supplementary.pdf]

Table S1. Comparison of the correlations between various biomarkers related with smoking exposure

Urinary biomarkers		Cotinine	Nicotine	Nornicotine	3-OH cotinine	Anabasine	TNE3
Cotinine	r		0.9106	0.8994	0.9376	0.6674	0.9630
	P value		< 0.0001	< 0.0001	< 0.0001	< 0.0001	< 0.0001
Nicotine		0.9106		0.9145	0.9022	0.6835	0.9416
		< 0.0001		< 0.0001	< 0.0001	< 0.0001	< 0.0001
Nornicotine		0.8994	0.9145		0.9245	0.7319	0.8711
		< 0.0001	< 0.0001		< 0.0001	< 0.0001	< 0.0001
3-OH cotinine		0.9376	0.9022	0.9245		0.6798	0.9282
		< 0.0001	< 0.0001	< 0.0001		< 0.0001	< 0.0001
Anabasine		0.6674	0.6835	0.7319	0.6798		0.6514
		< 0.0001	< 0.0001	< 0.0001	< 0.0001		< 0.0001
TNE3		0.9630	0.9416	0.8711	0.9282	0.6514	
		< 0.0001	< 0.0001	< 0.0001	< 0.0001	< 0.0001	

3-OH cotinine, trans-3′-hydroxycotinine; TNE3, total nicotine equivalents three (the molar sum of nicotine, cotinine, and 3-OH cotinine).

Table S2. Agreement of the classification of smoking status based on the biomarkers between single uri-nary cotinine and multiple nicotine metabolites

Biomarkers	Positive	Difference	Cohen's kappa
Cotinine alone	18.5%<br>(3,682/19.894)		
Cotinine or Nicotine	18.6%<br>(3,705/19,894)	0.1%<br>23/19,894	0.992<br>(0.990-0.995)
Cotinine or Nornicotine	18.6%<br>(3,699/19,894)	0.1%<br>17/19,894	0.994<br>(0.992-0.996)
Cotinine or 3-OH cotinine	18.8%<br>(3,737/19,894)	0.3%<br>55/19,894	0.982<br>(0.979-0.985)

3-OH cotinine, trans-3′-hydroxycotinine

20ng/mL for nicotine and cotinine, 50ng.mL for 3-OH cotinine, and 2 ng/mL for nornicotine as the cut-off values of four biomarkers for distinguishing between current smokers and non-smokers.

Table S3. Discrepancies between anabasine and nicotine metabolites without detectable

Cases	Number	Not detected (n, %)			
		Cotinine	Nicotine	Nornicotine	3-OH cotinine
Detected	4,286	1,205 (28.1%)	1,208 (28.2%)	1,329 (31.0%)	1,273 (29.7%)
Positive	3,566	856 (24.0%)	848 (23.8%)	927 (26.0%)	897 (25.2%)

3-OH cotinine, trans-3′-hydroxycotinine

20ng/mL for nicotine and cotinine, 50ng.mL for 3-OH cotinine, and 2 ng/mL for nornicotine and anabasine as the cut-off values of five biomarkers for distinguishing between current smokers and non-smokers.

Table S4. Analytical performances validation for five urinary biomarkers related with smoking exposure

Urinary biomarker	Precision (% CV)		Accuracy (% bias)		LLMI (ng/mL)	Linearity range (ng/mL)	R^2	Recovery (%)	Matrix effect (%RSD)
	Within-run ($n = 5$)	Between-run ($n = 30$)	Within-run	Between-run					
Cotinine	2.9-6.5	1.8-9.8	−5.6-0.8	−6.0-1.9	1	1-5,000	0.998	92.4-105.0	7.4-8.2
Nicotine	3.9-7.7	1.5-9.3	−5.8-−1.6	−7.6-1.5	1	1-10,000	0.999	90.3-102.9	5.4-6.9
Nornicotine	3.6-6.7	3.2-8.7	−6.3-0.3	−4.0-4.7	1	1-10,000	0.998	93.5-106.0	5.4-5.4
3-OH cotinine	3.3-5.2	4.7-9.8	−0.4-3.0	−8.5-3.1	5	5-15,000	0.999	94.2-109.0	4.2-7.6
Anabasine	2.9-9.4	2.4-8.6	−10.1-5.3	−5.0-2.8	1	1-10,000	0.999	86.8-103.4	4.3-10.1

3-OH cotinine, trans-3′-hydroxycotinine; LLMI, lower limit of measuring interval; RSD, Relative standard deviation

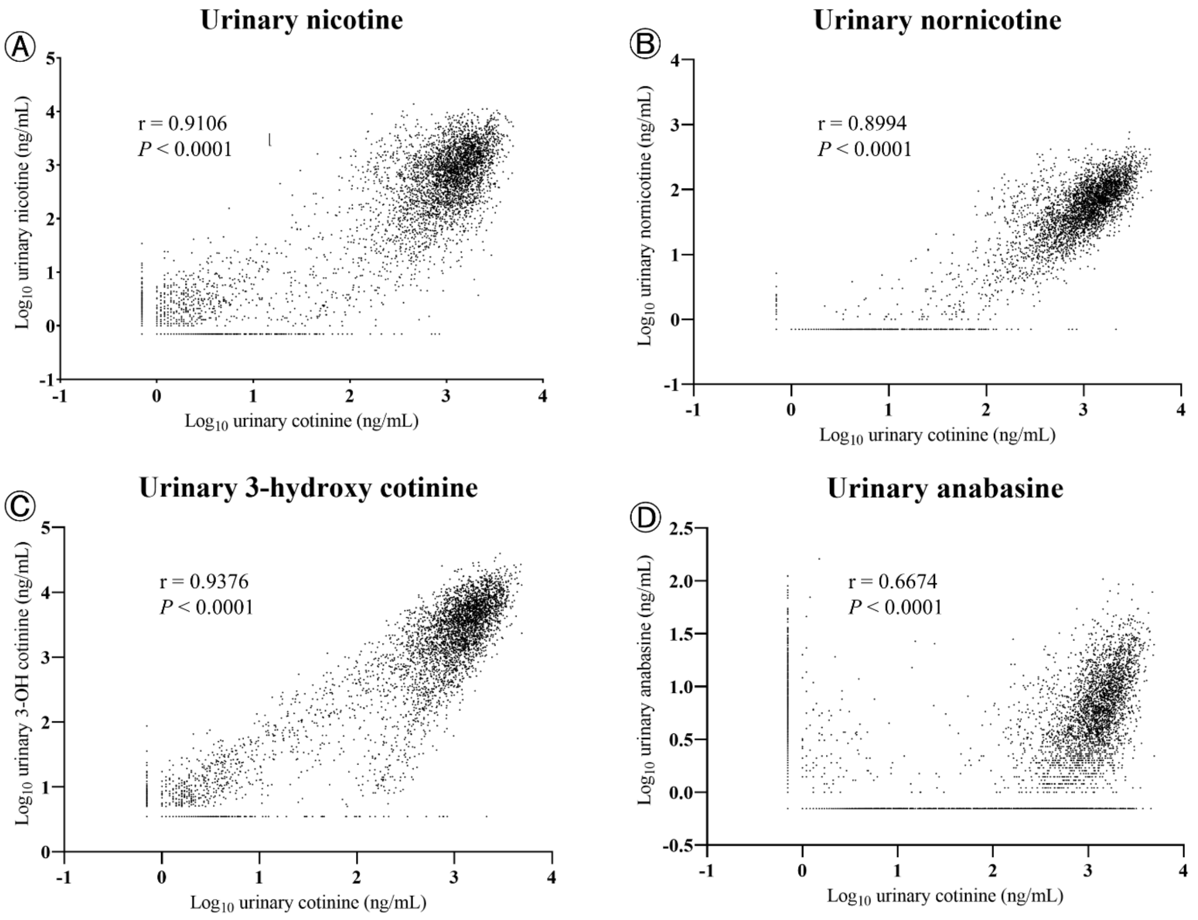

Figure S1.

The scatter plots of log-transformed urinary cotinine and various biomarkers related with smoking exposure

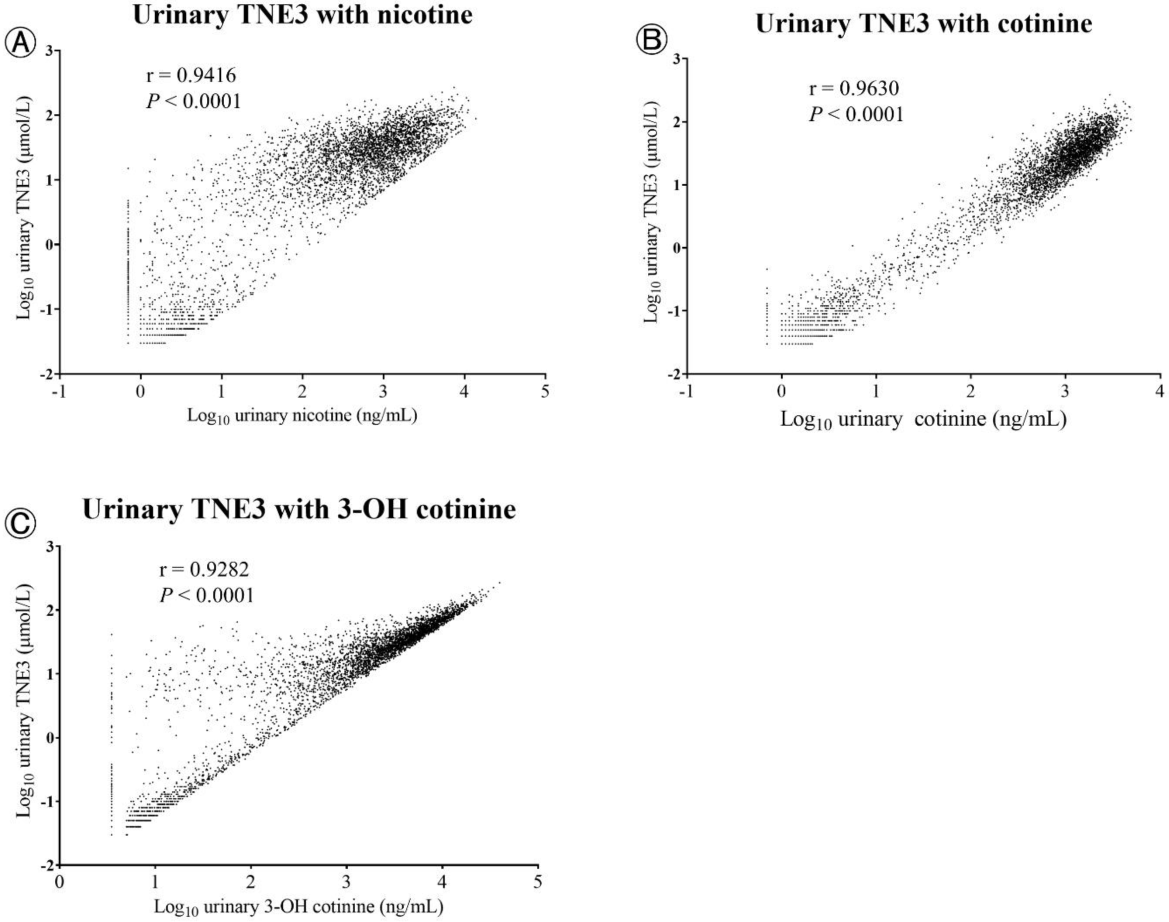

Figure S2.

The scatter plots of log-transformed urinary TNE3 and nicotine metabolites

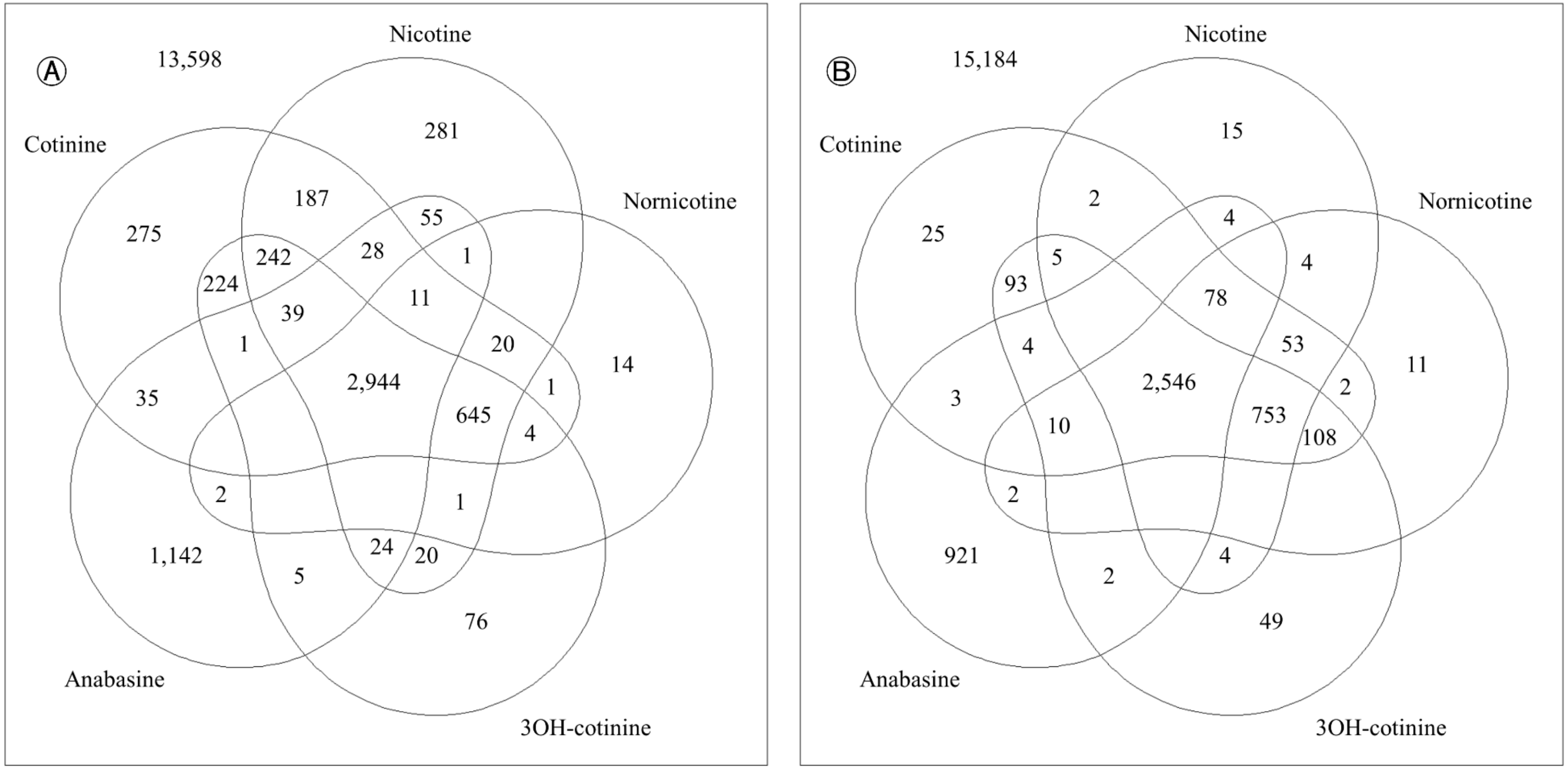

Figure S3.

Venn diagrams of five biomarkers for detection status (A) and classification of smoking status (B)

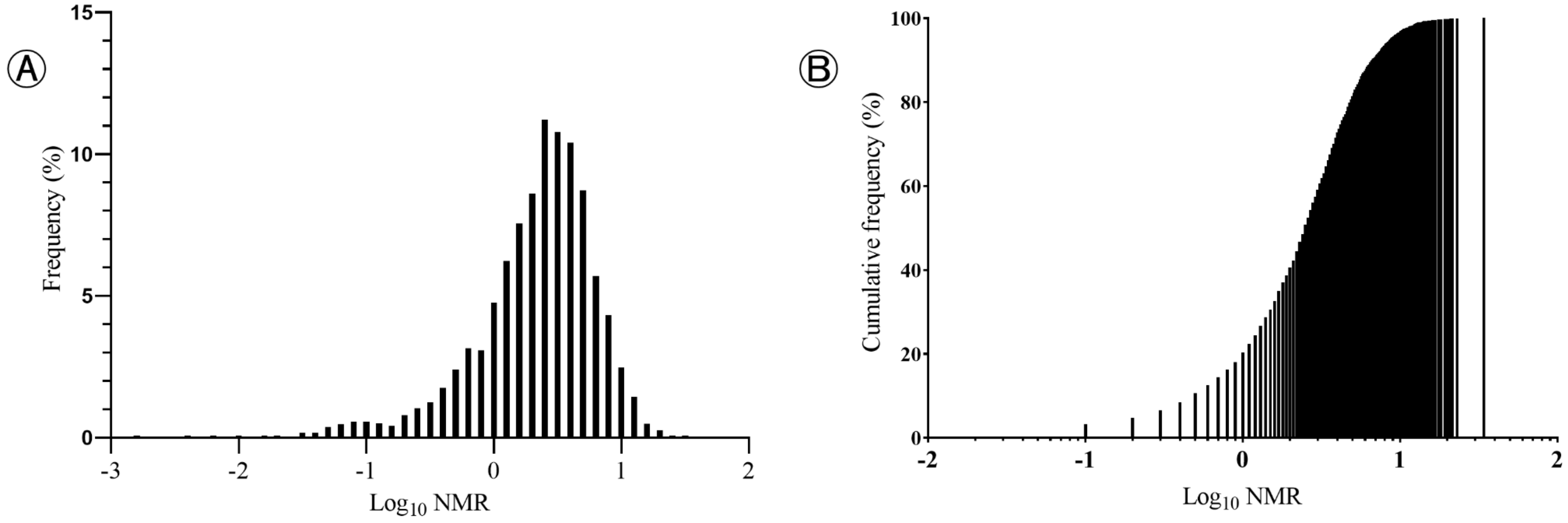

Figure S4.

Distribution of the ratio of urinary 3-OH cotinine to cotinine (NMR)

Frequency of log-transformed NMR (A) and its cumulative frequency (B)
